# A20 (*Tnfaip3*) Deficiency in Myeloid Cells Protects against Influenza A Virus Infection

**DOI:** 10.1371/journal.ppat.1002570

**Published:** 2012-03-01

**Authors:** Jonathan Maelfait, Kenny Roose, Pieter Bogaert, Mozes Sze, Xavier Saelens, Manolis Pasparakis, Isabelle Carpentier, Geert van Loo, Rudi Beyaert

**Affiliations:** 1 Unit of Molecular Signal Transduction in Inflammation, Department for Molecular Biomedical Research, Ghent, Belgium; 2 Department of Biomedical Molecular Biology, Ghent University, Ghent, Belgium; 3 Unit of Molecular Virology, Department for Molecular Biomedical Research, Ghent, Belgium; 4 Cell Culture and Sorting Core Facility, Department for Molecular Biomedical Research, Ghent, Belgium; 5 Institute for Genetics, University of Cologne, Cologne, Germany; Johns Hopkins University - Bloomberg School of Public Health, United States of America

## Abstract

The innate immune response provides the first line of defense against viruses and other pathogens by responding to specific microbial molecules. Influenza A virus (IAV) produces double-stranded RNA as an intermediate during the replication life cycle, which activates the intracellular pathogen recognition receptor RIG-I and induces the production of proinflammatory cytokines and antiviral interferon. Understanding the mechanisms that regulate innate immune responses to IAV and other viruses is of key importance to develop novel therapeutic strategies. Here we used myeloid cell specific A20 knockout mice to examine the role of the ubiquitin-editing protein A20 in the response of myeloid cells to IAV infection. A20 deficient macrophages were hyperresponsive to double stranded RNA and IAV infection, as illustrated by enhanced NF-κB and IRF3 activation, concomitant with increased production of proinflammatory cytokines, chemokines and type I interferon. *In vivo* this was associated with an increased number of alveolar macrophages and neutrophils in the lungs of IAV infected mice. Surprisingly, myeloid cell specific A20 knockout mice are protected against lethal IAV infection. These results challenge the general belief that an excessive host proinflammatory response is associated with IAV-induced lethality, and suggest that under certain conditions inhibition of A20 might be of interest in the management of IAV infections.

## Author Summary

Influenza virus or flu epidemics represent a recurrent threat to the public health, especially for individuals which are part of a high-risk group such as children, elderly or immune-compromised people. Sporadic pandemic flu outbreaks, such as the Spanish flu of 1918, may cause high grades of mortality among healthy persons. A better understanding of how the immune system deals with these pathogens is of key importance. The protein A20 is an important negative regulator of both innate and adaptive immune responses. We show that the specific deletion of *A20* in myeloid cells, such as macrophages and neutrophils, improves the resistance against otherwise lethal influenza infections. This protective effect is mediated by an enhanced innate immune response following respiratory challenge with influenza virus. Although exaggerated pulmonary immune responses are believed to be the primary cause of often life threatening influenza virus induced pneumonia, we demonstrate that boosting the innate immune response by selectively targeting the functionality of A20 in myeloid cells is beneficial for the host survival. This finding provides us with a novel valuable approach for treating influenza and potentially other respiratory viral infections.

## Introduction

Viruses are a class of highly diverse pathogens which depend on the host cell for their replication. The initiation of a protective innate antiviral immune response involves the action of specialized pattern recognition receptors (PRR), which detect conserved molecular structures of the invading pathogen. Triggering of PRRs induces the production of host proinflammatory cytokines (e.g. TNF, IL-6 and IL-1) and type I interferons (interferon-α (IFN-α) and IFN-β) through activation of downstream signaling pathways that control various transcription factors such as NF-κB, AP-1, IRF3 and IRF7 [1], [2]. The presence of viral nucleic acids, such as viral RNA and DNA, viral replication intermediates and viral transcription products, can be sensed by specific intracellular PRRs [3]. Endosomal Toll-like receptors (TLRs) and cytoplasmic RNA helicase RIG-I-like receptors (RLRs) or Nod-like receptors (NLRs) detect the presence of viral single stranded (TLR7, TLR8, Nod2) or double stranded RNA (TLR3, RIG-I, MDA5). Intracellular DNA sensors that mediate antiviral immune responses to DNA viruses include TLR9, DAI [4] and the PYHIN domain containing proteins AIM2 [5], [6], [7] and IFI16 [8]. TLR mediated antiviral responses are restricted to specialized type I IFN producing plasmacytoid dendritic cells (pDC), while most other cell types, including conventional DC (cDC), macrophages and fibroblasts, depend on the cytosolic RNA and DNA sensors for the production of antiviral proteins [9].

Influenza A virus (IAV) is the etiological agent of a contagious acute respiratory disease that causes considerable mortality, which is generally believed to be due to an excessive host inflammatory response. Emergence of drug-resistant strains of influenza viruses with pandemic potential underscores the importance of developing novel antiviral strategies. In this context, understanding of the mechanisms that regulate IAV-induced immune responses is critical. IAV infection leads to the exposure in the host cell of single-stranded genomic RNA and double stranded RNA, the latter being an intermediate of viral replication. TLR3 and RIG-I have been implicated as sensors of IAV infection [10]–[12]. Both receptors contribute to the proinflammatory response to IAV, but the initiation of the innate antiviral immune response largely depends on RIG-I mediated signaling [13]. Interestingly, RIG-I deficient mice are highly susceptible to IAV [14], [15], whereas TLR3 deficient mice have a survival advantage to acute infection [16]. These results indicate that an imbalance between the beneficial and harmful effects of mediators released by immune cells is likely to contribute to the pathogenesis of influenza.

RIG-I contains a C-terminal DExD/H box helicase domain, which is required for ligand recognition, and two N-terminal CARD domains. Upon ligand binding, the CARD domains of RIG-I associate with the CARD domain of the MAVS (also termed IPS-1, VISA, Cardif) adaptor protein, which subsequently translocates to and inserts in the outer mitochondrial membrane via its C-terminal transmembrane domain [17]–[20]. Signaling downstream of MAVS requires the action of various ubiquitin modifying enzymes, which both positively and negatively regulate RIG-I mediated signal transduction [21]. K63-specific ubiquitin ligases (E3s), such as TRIM25 [22] and Riplet [23], [24], have been shown to directly promote RIG-I activation. In addition, well characterized ubiquitin ligases such as TRAF6 [25], [26] and TRAF3 [27] mediate respectively NF-κB and IRF3 activation upon RIG-I stimulation. On the other hand, deubiquitinating enzymes (DUBs), such as DUBA [28], CYLD [29], [30] and OTUB1/2 [31] have been shown to negatively regulate RLR signaling by specifically removing K63-linked polyubiquitin chains from several signaling molecules. Furthermore, various K48-specific ubiquitin ligases, such as AIP4 [32] and TRIAD3A [33] mark respectively MAVS and TRAF3 for proteasome mediated degradation, thus inhibiting further downstream signaling. Additionally, the attachment of K48-specific polyubiquitin chains to the IRF3 and IRF7 transcription factors by E3s such as RAUL [34], TRIM21 [35] and RBCK1 [36] further dampens antiviral signal transduction.

A20 is an ubiquitin-editing enzyme belonging to the OTU-domain family of DUBs. Interestingly, A20 also harbors atypical zinc finger dependent K48-specific E3 ubiquitin ligase activity. Both catalytic and noncatalytic mechanisms were previously shown to be involved in the negative regulation of proinflammatory signaling by A20 in response to multiple receptors such as TNF receptor I [37]–[39], TLR4 [40], and IL-1R [41]. The anti-inflammatory role of A20 is clearly demonstrated by the fact that A20 deficient mice die early after birth due to severe multi-organ inflammation and cachexia [37]. More recently, gene targeting of A20 in specific cell types was shown to be associated with autoimmunity and chronic inflammation [42]–[48], further illustrating that A20 is an important brake on the inflammatory response. The relevance of these findings for human disease has recently been illustrated through the identification of polymorphisms in the *A20* locus that are associated with several autoimmune diseases and chronic inflammation [45]. In contrast to its well established function in the regulation of proinflammatory responses, the role of A20 in the regulation of antiviral immune responses is less well described and limited to a number of *in vitro* studies using overexpression or silencing in specific cell lines, indicating that A20 may regulate RIG-I- and TLR3-induced signaling to NF-κB and IRF-3 [49]–[52]. However, the precise *in vivo* role of A20 in the response to viral infection remains to be clarified. Using myeloid cell specific A20 knockout mice (A20^myel-KO^) that were recently generated in our lab and primary cells derived of these mice, we here provide evidence that A20 is a crucial negative regulator of IAV-induced proinflammatory and antiviral signaling in macrophages. Interestingly, A20^myel-KO^ mice show enhanced survival and reduced morbidity in response to IAV lung infection compared to wild type mice. Protection against IAV in A20^myel-KO^ mice is associated with increased cytokine and chemokine production, augmented recruitment of innate immune cells such as neutophils and alveolar macrophages, and enhanced viral clearance. These results suggest that boosting the innate immune response to IAV by targeting A20 activity in myeloid cells might have therapeutic potential.

## Results

### A20 inhibits RIG-I-induced NF-κB and IRF3 activation

**Figure 1:**
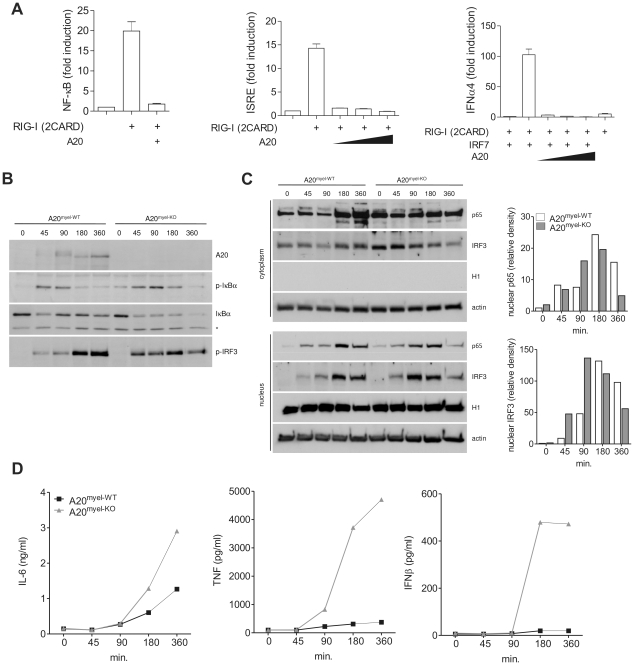
A20 inhibits NF-κB and IRF3 activation in response to RIG-I stimulation. (A) HEK293T cells were transfected with NF-κB (left), IRF3 (middle) or IRF7 dependent luciferase reporter (right) plasmids, together with plasmids expressing RIG-I (2CARD), IRF7 (right) and increasing amounts of A20. Luciferase activity in cell extracts was analyzed in a luminometer. Numbers are averages +/− SD of 3 samples per set-up. (B–D) A20^myel-KO^ and wild type (A20^myel-WT^) BMDM were transfected with LMW poly(I:C) and analyzed at different time points (minutes) after the start of transfection for IκBα phosphorylation and degradation, and IRF3 phosphorylation by immunoblotting of total cell extracts (B). The band that is non-specifically recognized by the anti-IκBα antibody (indicated by an asterisk) can be used as a loading control. Nuclear translocation of NF-κB (p65) and IRF3 was analyzed by immunoblotting of cytoplasmic or nuclear fractions (C). Quantification of band density for nuclear p65 and IRF3 is shown in bar graphs (expressed as relative density compared to the first lane;  = time 0). IL-6, TNF and IFNβ levels in the supernatant of cells used in (B) were determined by bioassay (IL-6) and ELISA (TNF and IFNβ) as described in the ‘materials and methods’ section (D). Data are representative of 2 independent experiments.

RIG-I signaling induces the activation of NF-κB, IRF3 and IRF7 transcription factors, which promote the expression of proinflammatory cytokines and type I IFNs that restrict further viral propagation. Previous studies have shown that ectopically expressed A20 negatively regulates NF-κB and IRF3 activation upon RIG-I stimulation [50]–[52]. Similarly, we show that A20 overexpression in HEK293T cells prevents NF-κB- and IRF3-dependent luciferase reporter gene activation induced by transfection of a truncated constitutive active form of RIG-I [53], corresponding to only the two N-terminal CARD domains of RIG-I [RIG-I (2CARD)] (figure 1A, left and middle graph). We next investigated whether A20 also inhibits IRF7 activation. Unlike IRF3, IRF7 is not or weakly expressed under naïve conditions and IRF7 protein levels are rapidly upregulated upon virus-induced IRF3 activation [54], [55]. To determine the effect of A20 on IRF7 activation, we therefore transfected minor amounts of an IRF7 expression plasmid together with plasmids encoding RIG-I (2CARD), A20 and an IRF7-specific IFNα4 luciferase reporter construct. RIG-I (2CARD) expression in the absence of IRF7 co-expression showed negligible IFNα4 promoter activation (grey bar, figure 1A, right graph), whereas significant reporter gene expression was observed in the presence of IRF7. Similar to its inhibitory effect on NF-κB and IRF3 activation, A20 also prevented RIG-I-induced IRF7 activation (figure 1A, right graph). These results demonstrate the potential of A20 to inhibit RIG-I-induced NF-κB and IRF3/7 activation.

To study the effect of endogenously expressed A20 on RIG-I-induced signaling in a more immunological relevant context, we performed further experiments in A20 deficient primary macrophages. Since A20 full knockout mice die prematurely as a result of severe multi-organ inflammation [37], we generated mice carrying a conditional A20 allele in which exon IV and exon V were flanked by two loxP sites [56]. Crossing these mice with transgenic mice expressing Cre recombinase under control of the lysozyme M promoter leads to specific deletion in myeloid cells and allowed us to generate myeloid cell specific A20 knockout mice [48]. To stimulate the RIG-I receptor, we transfected A20^myel-KO^ BMDM and wild type control cells with minimal amounts of low molecular weight (LMW) poly(I:C), which is known to preferentially bind and activate RIG-I rather than MDA5 [57]. Of note, this concentration of poly(I:C) was unable to induce significant TLR3 dependent NF-κB and IRF3 activation or cytokine production (data not shown). As expected, poly(I:C) transfection induced the rapid expression of A20 in wild type, but not in A20^myel-KO^ BMDM (figure 1B, upper panel). At early time points, slightly slower migrating forms of A20 were observed, indicating that A20 undergoes a yet unknown modification upon poly(I:C) transfection. Compared to wild type BMBM, A20 deficient cells showed enhanced NF-κB activation as indicated by increased phosphorylation and sustained degradation of IκBα (figure 1B). Furthermore, nuclear translocation of the p65 NF-κB subunit was enhanced in poly(I:C) transfected A20^myel-KO^ BMDM, reaching a maximum at earlier time points compared to wild type cells (figure 1C). IRF3 is known to be activated upon phosphorylation of a series of carboxyl terminal serine residues by the IKK-like kinases TBK1 and IKK_ε_ [58], leading to its dimerization and subsequent translocation to the nucleus [59]. Using immunoblotting with an antibody directed against phosphorylated Ser396, maximum IRF3 phosphorylation was detected at earlier time points in A20^myel-KO^ BMDM compared to wild type BMDM (figure 1B). Similar to p65, IRF3 nuclear translocation reached its maximum at an earlier time point in A20 deficient BMDM compared to wild type cells (figure 1C). NF-κB controls the expression of IL-6 and TNF, and NF-κB and IRF3 control the expression of IFNβ. In line with the enhanced activation of NF-κB and IRF3 as described above, A20^myel-KO^ BMDM secreted increased amounts of IL-6, TNF and IFNβ (figure 1D). Similar results were obtained using peritoneal macrophages (data not shown). Together, these results demonstrate that A20 plays an important role in the negative regulation of RIG-I-induced NF-κB and IRF3 activation in primary macrophages.

### A20 negatively regulates IAV-induced gene expression in BMDM

**Figure 2:**
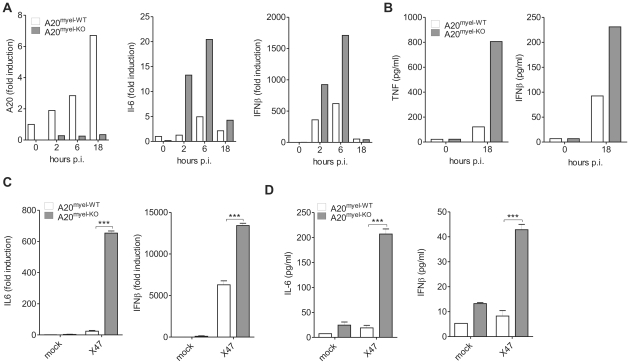
A20 negatively regulates IAV-induced gene expression in BMDM and alveolar macrophages. (A–B) BMDM isolated from A20^myel-WT^ and A20^myel-KO^ animals were infected with IAV (moi 1). At different hours post infection (hours p.i.) cells were lysed and IL-6, IFNβ and A20 mRNA expression was analyzed by qPCR (A). 18 hours post infection cell culture supernatant was analyzed for TNF and IFNβ protein levels by ELISA and multiplexing technologies (B). (C–D) Alveolar macrophages were mock treated or infected with IAV (moi 1) for 18 hours. IL-6 and IFNβ mRNA expression was determined by qPCR (C). Cell culture supernatant was analyzed for IL-6 and IFNβ protein levels by ELISA and multiplexing technologies (D). Error bars represent mean values (+/− SEM) of 2–3 samples. Results are representative for 2 independent experiments. ***p<0,001.

To investigate the role of A20 in the IAV-induced proinflammatory and antiviral innate immune responses, we infected A20 deficient and control BMDM with IAV X-47 (H3N2). A20 mRNA levels were rapidly induced in wild type BMDM, but not in A20 deficient BMDM, upon viral infection (figure 2A). Furthermore, A20^myel-KO^ BMDM show enhanced expression of IL-6 and IFNβ mRNA after IAV infection compared to control cells (figure 2A). In accordance with these data, cell culture supernatant collected from these cells contained higher levels of TNF and IFNβ (figure 2B).

Upon host infection with IAV, alveolar macrophages are an important source of cytokines and chemokines, attracting innate immune cells to the lung during the primary phase of infection. To test whether A20 directly controls IAV-induced gene expression in alveolar macrophages, we isolated these cells from lungs of A20^myel-KO^ and control littermates and infected them *in vitro* with IAV X-47. Expression and secretion of the proinflammatory cytokines IL-6 and TNF, the type-I IFN IFNβ and IFNα4 and the chemokines MCP-1 (ccl2) and KC (cxcl1) was significantly higher in IAV infected cells lacking A20 compared to infected wild type cells (figure 2C and figure S1). Taken together these results demonstrate that A20 negatively regulates IAV-induced proinflammatory and antiviral gene expression in alveolar macrophages, consistent with the inhibitory effect of A20 seen on RIG-I-induced NF-κB and IRF3 activation.

### A20 deficiency in myeloid cells protects against IAV lung infection

**Figure 3:**
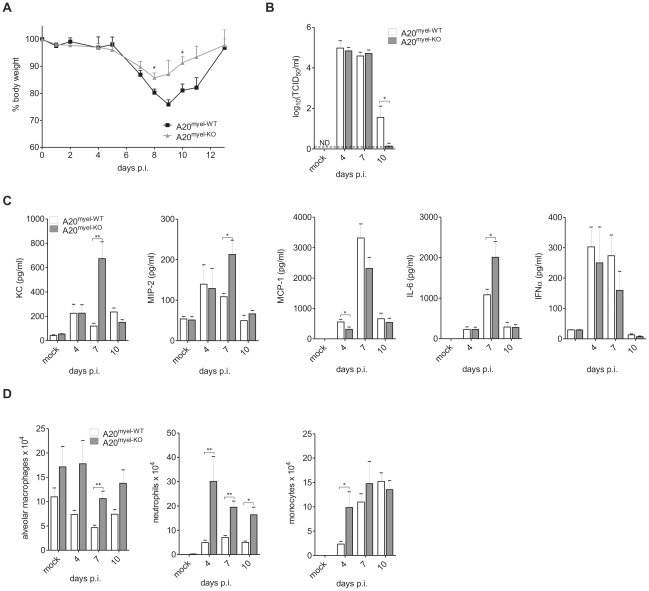
A20 deficiency in myeloid cells protects against IAV lung infection. A20^myel-WT^ and A20^myel-KO^ mice were infected intranasally with a sublethal dose of IAV and weight loss was monitored (A). At day 4, 7 and 10 post infection (p.i.) viral titers in the lung were measured and expressed as mean TCID_50_ (B). The dashed line represents the detection limit. ND, not detectable. BAL was isolated from IAV infected mice at 4, 7 and 10 days p.i. and KC, MIP-2, MCP-1, IL-6 and IFNα protein levels were analyzed by ELISA and multiplexing technologies (C). Absolute numbers of resident alveolar macrophages, neutrophils and monocytes in BAL were analyzed by flow cytometry at 4, 7 and 10 days p.i. (D). Numbers are averages +/− SEM of at least 5 mice per group and are representative of 2 independent experiments. *p<0.05; **p<0.01.

To determine the role of A20 expression in myeloid cells during an IAV infection *in vivo*, we intranasally inoculated both A20^myel-KO^ mice and control littermates with a sublethal dose of the mouse adapted IAV X-47 (H3N2) strain and monitored morbidity in terms of weight loss. A20^myel-KO^ mice showed reduced weight loss compared to wild type control littermates and recovered faster from the viral challenge (figure 3A). Also, total protein concentration in BAL fluid, which reflects lung damage and vascular leakage, was increased significantly at 7 and 10 days post infection in both wild type and A20^myel-KO^ mice, and was slightly lower in A20^myel-KO^ mice (data not shown). Next, we measured pulmonary viral titers at 4, 7 and 10 days post infection. No differences in viral titers were observed in A20^myel-KO^ mice versus wild type mice at day 4 and 7 post infection. However, after 10 days, almost no virus could be detected in the lungs of A20^myel-KO^ mice while abundant infectious viral particles could still be isolated from lungs of all wild type mice (figure 3B). This indicates that loss of A20 in myeloid cells does not affect early viral replication but contributes to viral clearance at later stages during infection.

To verify if A20 deficiency in myeloid cells affects IAV-induced gene expression in the lung, we analyzed the levels of several chemokines and cytokines in the bronchoalveolar lavage (BAL) at day 4, 7 and day 10 following infection. Levels of KC and MIP-2 chemokines, as well as IL-6 were significantly higher at day 7 p.i. in BAL from IAV infected A20^myel-KO^ mice compared to IAV infected wild type mice (figure 3C). Unlike our observations with *in vitro* stimulated primary macrophages we could not detect a significant increase in MCP-1 or IFNα production in the lungs of A20^myel-KO^ animals (figure 3C). KC is the murine orthologue of IL-8 and serves together with MIP-2 as a chemoattractant for neutrophils, while MCP-1 is mainly known as a chemoattractant for monocytes, which eventually develop into macrophages upon entering the alveolar lumen [60]. Consistent with the higher KC and MIP-2 levels in A20^myel-KO^ mice, the number of neutrophils (CD11b^+^ Ly6C^+^ Ly6G^+^ F4/80^−^) that were recruited in the bronchoalveolar spaces upon IAV infection was clearly higher throughout infection in A20^myel-KO^ mice compared to control animals (figure 3D). Although we could detect a significant increase in monocyte (CD11b^+^ Ly6C^+^) recruitment at day 4 post infection, this was not evident at later time points after infection (figure 3D), which is consistent with the equal expression of MCP-1 in both groups of mice. The number of resident alveolar macrophages (autofluorescent^+^ CD11c^+^ F4/80^+^ CD11b^−^) was also elevated in A20^myel-KO^ mice but did not differ significantly between IAV infected or mock infected mice (figure 3D). Elimination of IAV infected cells depends on the clonal expansion of virus specific cytotoxic CD8^+^ T cells (CTL) [61], [62], [63]. To test whether A20 expression in myeloid cells regulates the antiviral CTL response, total CD8^+^ T cells and virus specific Granzyme B (GrB) and IFNγ expressing CD8^+^ T cells were measured in BAL and lung parenchyma of wild type and A20^myel-KO^ mice. A clear increase in CD8^+^ T cells could be detected at day 7 and 10 post infection, but no differences were observed between A20^myel-KO^ and wild type mice (figure S2A and figure S2C). Also, the number of GrB and IFNγ CD8^+^ T cells as well as IFNγ expression levels in the lungs were not altered by the absence of A20 in myeloid cells (figure S2A–C).

Protection against IAV infection is also provided by the humoral immune response. To test whether loss of A20 in myeloid cells affects B cell mediated immunity, we determined hemagglutinin (HA) antibody titers in the serum of A20^myel-KO^ and wild type littermates. However, no differences could be detected between wild type and A20^myel-KO^ animals (figure S2D), indicating that humoral immunity is not affected by A20 expression in myeloid cells. Together, these data suggest that mechanisms other than adaptive immunity, such as an increased innate immune response, characterized by an increased influx of neutrophils and increased numbers of alveolar macrophages, contribute to the better viral clearance in A20^myel-KO^ mice.

**Figure 4:**
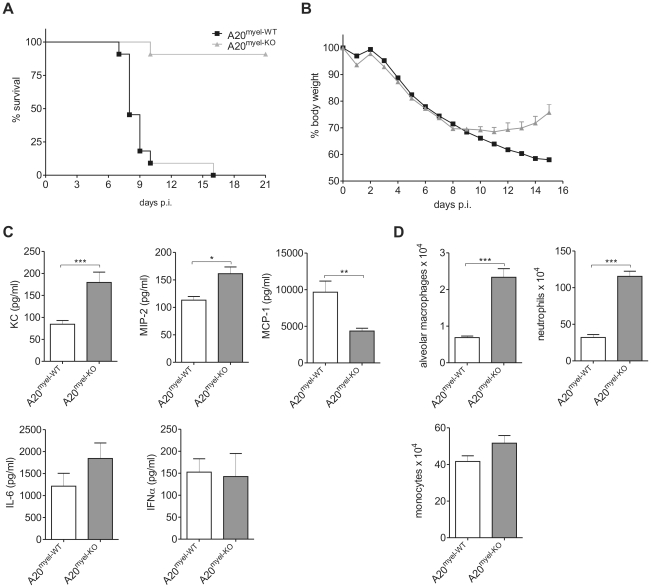
A20 deficiency in myeloid cells protects against lethal IAV infection. A20^myel-WT^ (n = 11) and A20^myel-KO^ (n = 11) mice were intranasally infected with a lethal dose of IAV and survival (p<0.001) (A) and weight loss +/− SEM (B) was monitored for respectively 21 and 15 days p.i. BAL fluid was collected from wild type (A20^myel-WT^) and A20^myel-KO^ mice 6 days following infection with a lethal dose IAV and KC, MIP-2, MCP-1, IL-6 and IFNα protein levels were determined (C). Absolute numbers of alveolar macrophages, neutrophils and monocytes in BAL were analyzed by flow cytometry at 6 days p.i. (D). Numbers are averages +/− SEM of at least 5 mice per group. Data are representative of 2 independent experiments. *p<0.05; **p<0.01, ***p<0.001.

It is generally believed that IAV-induced mortality is due to an excessive proinflammatory response in the lung. We therefore analyzed whether the increased proinflammatory cytokine production and infiltration of proinflammatory cells in A20^myel-KO^ mice affects mortality induced by intranasal infection with a lethal dose of IAV X-47. Surprisingly, almost all A20^myel-KO^ mice survived (10/11), while all control mice succumbed (0/11) within 16 days after infection (figure 4A). A20^myel-KO^ mice still showed significant weight loss and lung damage (as reflected by increased total protein concentration in the BAL; data not shown) during the course of infection but were able to recover, in contrast to wild type mice that succumbed (figure 4B). Similar to our observations with sublethal IAV infection, pulmonary KC and MIP-2 production was stronger in A20^myel-KO^ animals compared to wild type mice following lethal IAV infection (figure 4C), which correlates with the increased numbers of neutrophils in the lungs of these mice (figure 4C). Also levels of the proinflammatory cytokines IL-6, TNF and IL-1β, which are often associated with immunopathogenesis in humans [64], were increased in the lungs of A20^myel-KO^ mice compared to control animals (figure 4C and figure S3A). Again, MCP-1 production was not increased and even lower in A20^myel-KO^ mice (figure 4C), and also monocyte recruitment was not different between both groups of mice. We could also not detect any differences in viral clearance or antiviral adaptive immunity at 6 h post infection (figure S3B–F). Collectively these data indicate that A20 deficiency in myeloid cells is associated with an increased innate immune response and protection against a lethal IAV infection.

## Discussion

In the present study we have investigated the contribution of A20 expression in myeloid cells in the innate immune response to IAV lung infection. In the pulmonary environment, macrophages populate both lung parenchyma and the alveolar lumen where they are referred to as alveolar macrophages. Under naïve conditions, alveolar macrophages exert important immunomodulatory functions [65], [66]. However, alveolar macrophages are also crucial in the innate immune response against IAV as they are one of the first cells that encounter infectious IAV particles [67], [68]. They are an important source of proinflammatory cytokines and chemokines that attract innate immune cells to the lung during the primary phase of infection [69], and they are the primary producers of type I IFNs [70]. Alveolar macrophages are also known to phagocytose virus infected apoptotic cells and antibody coated viral particles, further contributing to viral clearance. We could show that BMDM or alveolar macrophages derived from A20^myel-KO^ mice express higher amounts of chemokines, cytokines and type I IFNs compared to wild type mice in response to *in vitro* infection. Similarly, *in vivo* infection with IAV resulted in higher levels of primarily neutrophil attracting chemokines such as KC and MIP-2 and several proinflammatory cytokines such as IL-6, TNF and IL-1β in the lungs of A20^myel-KO^ mice compared to wild type mice. This was associated with a selective enhancement of neutrophil recruitment to the bronchoalveolar compartment, and resulted in improved viral clearance later on during infection. Although the role of neutrophils during viral infection is still under debate, recent evidence supports a protective function of these cells during IAV infection [71], [72]. MCP-1 levels were not affected by the absence of A20 in myeloid cells, which is consistent with the notion that airway epithelial cells are the primary source of MCP-1 production during IAV infection [73]. Mice deficient for the MCP-1 receptor CCR2, which is expressed on a subset of circulating monocytes, are protected against IAV infection and display reduced signs of immunopathology [74]–[76]. During IAV infection these monocytes develop into inflammatory dendritic cells or proinflammatory macrophages [77] and are considered major contributors to IAV-induced immunopathology [78]. A20^myel-KO^ mice were protected against a lethal IAV infection, which is rather surprising since an excessive proinflammatory cytokine response and an excessive influx of inflammatory cells is generally believed to be associated with increased lethality [64], [79]. However, the selective effect of A20 deficiency on neutrophil recruitment, without altering inflammatory monocyte (Ly6C^+^ CD11b^+^) recruitment, further support the idea that monocytes and not neutrophils are major contributors to IAV-associated immunopathology and lethality [78].

We show that A20 deficient BMDM display enhanced NF-κB and IRF3 activation in response to RIG-I stimulation by synthetic LMW double stranded RNA. RIG-I has previously been shown to play a key role in the innate immune response to IAV [13], suggesting that the increased immune response of A20^myel-KO^ mice to IAV lung infection reflects enhanced RIG-I signaling. We propose that A20 inhibits IAV-induced proinflammatory gene expression (as shown in our manuscript for TNF, IL-6, KC, MIP-2, and IFNβ) by negatively regulating NF-κB and IRF3 activation, which are the major pathways controlling these genes. However, this does not exclude an additional effect of A20 on other signaling pathways that may contribute to proinflammatory gene expression. A20 is believed to exert its NF-κB and IRF3 inhibitory functions by modulating the ubiquitination status of different signaling proteins [80]. In this context, it was recently shown that A20 cooperates with the ubiquitin-binding proteins TAX1BP1 and ABIN1 to to disrupt the TRAF3-TBK1-IKKε complex, thereby negatively affecting K63-polyubiquitination of TBK1 and IKKε, and their ability to activity IRF3 [81], [82]. Whether similar mechanisms are involved in the regulation of RIG-I induced NF-κB activation is still unclear. So far we were unable to clearly detect ubiquitination of TBK1 and IKKε in primary macrophages, preventing us from studying the effect of A20 deficiency on their ubiquitination status. It cannot be excluded however that A20 also targets other substrates that mediate NF-κB and IRF3 activation in myeloid cells. The identification of these substrates will be the topic of future investigations in our laboratory. Multiple other deubiquitinating enzymes (DUBs), such as DUBA [28], CYLD [29], [30], OTUB1/2 [31], and A20 [49]–[52] have been shown to negatively regulate RIG-I signaling to NF-κB and IRF-3, implicating possible redundancy. However, evidence so far was limited to *in vitro* data and was obtained under non-physiological conditions. The clear protective phenotype of A20^myel-KO^ mice that we here describe indicates that A20 expression in myeloid cells is a central gatekeeper of RIG-I induced signaling in response to IAV infection and that other DUBs cannot substitute for A20 deficiency under physiological conditions. If A20 has a similar non-redundant role in other cell types that are implicated in the response to IAV, such as lung epithelial cells, remains to be investigated.

Understanding and controlling the activation of innate antiviral immune responses is an important step toward novel therapies. About a fifth of world's population is infected by IAV annually, leading to high morbidity and mortality, particularly in infants, the elderly and the immunocompromised. The high mutation rate of IAV turns all attempts of vaccine and antiviral design into a never ending battle. In recent years, RNA interference, triggered by synthetic short interfering RNA (siRNA), has rapidly evolved as a potent antiviral regimen. Properly designed siRNAs have been shown to function as potent inhibitors of influenza virus replication. Although specificity and tissue delivery remain major bottlenecks in the clinical applications of RNAi in general, intranasal application of siRNA against respiratory viruses including, but not limited to influenza virus, has experienced significant success and optimism [83]. Our results suggest that not only siRNA targeting IAV components, but boosting the antiviral immune response by intranasal administration of siRNA against A20 might be a valid therapeutic approach. Also small compound inhibitors of A20 might be an interesting alternative. Finally, similar targeting of A20 might be of interest in the battle against other viral infections including RSV and SARS coronavirus.

## Materials and Methods

### Ethics statement

All experiments on mice were conducted according to the national (Belgian Law 14/08/1986 and 22/12/2003, Belgian Royal Decree 06/04/2010) and European (EU Directives 2010/63/EU, 86/609/EEG) animal regulations. Animal protocols were approved by the ethics committee of Ghent University (permit number LA1400091, approval ID 2010/001). All efforts were made to ameliorate suffering of animals. Mice were anesthetized by intraperitoneal (i.p.) injection of a mixture of ketamine (12 mg/kg) and xylazine (60 mg/kg).

### Mice

A20^fl/fl^ mice were generated as previously described [56]. A20^fl/fl^ mice were crossed with LysM-Cre mice [84] (provided by I. Förster, Institute of Genetics, University of Cologne, Germany) to generate A20^fl/fl^ LysMCre transgenes and are described in detail elsewhere [48]. Mice were housed in individually ventilated cages at the VIB Department of Molecular Biomedical Research in specific pathogen-free animal facilities. Influenza infections were performed on age- (between 7 and 9 weeks old) and sex-matched littermates. A20^fl/fl^ LysM-Cre animals were backcrossed three times to the C57Bl/6 background. A20^fl/fl^ mice expressing or lacking the LysM-Cre transgene were termed A20^myel-KO^ and wild type (A20^myel-WT^) respectively.

### Viral infection and determination of viral titers

Mouse adapted IAV X-47 (H3N2; PR8×A/Victoria/3/75) was propagated in MDCK cells. For viral inoculation, mice were anesthetized by i.p. injection with ketamine (12 mg/kg) and xylazine (60 mg/kg) and 50 µl X-47 diluted in PBS was administered intranasally. For lethal and sublethal infection, mice received respectively 2-LD_50_ or 0.05-LD_50_ X-47. To determine pulmonary viral titers, median tissue culture infectious dose (TCID_50_) was measured as follows: lungs were homogenized with a Polytron homogenizer (Kinematica) in PBS. Eight-fold serial dilutions of lung homogenates were incubated on MDCK cells for 5 days in DMEM supplemented with trypsin (1 µg/ml), 2 mM L-glutamine, 0.4 mM sodium pyruvate and antibiotics. For read-out, 0.5% chicken red blood cells (RBC) were added and end-point dilution of hemagglutination was monitored. TCID_50_ titers were then calculated according to the method of Reed and Muench [85].

### Determination of HAI (hemagglutination inhibition) titers

To determine the HAI titers in infected mice, sera of these were treated with receptor-destroying enzyme (RDE/Cholera filtrate; Sigma) to remove sialic acids from serum proteins capable of aspecific inhibition of agglutination. After incubation overnight at 37°C, the RDE was inactivated by addition of 0.75% sodium citrate in PBS and heating to 56°C for 30 min. To remove sialic acid binding proteins, sera were cleared with 1/10 volume 50% chicken RBC. Titration was done by incubating a two-fold dilution series of sera with 4 HA units of X-47 virus for 1 hour at room temperature in 96-well U-bottom plates. Finally, an equal volume of 0.5% chicken RBC was added and titers were read 30 min later. Negative controls included PBS instead of immune serum (agglutination control) or PR8 instead of X-47 virus (control for agglutination effect of sera); as positive control, serum from a mouse infected twice with a sublethal dose of X-47 was used.

### *In vivo* intracellular GrB and IFNγ staining of activated CD8+ T cells

Granzyme B (GrB) and IFNγ expressing CD8^+^ T cells were determined by treating the mice intranasally with 50 µg Brefeldin A (Sigma) as previously described [86]. 6 h later, BAL and lungs were isolated and single cell suspensions were prepared from the lung in the presence of 3 µg/ml Brefeldin A. Cells were stained, fixed and permeabilized (Cytofix/Cytoperm, BD Biosciences) according to the manufacturer's instructions. Activated CD8^+^ T cells were analyzed by flow cytometry based on CD62^lo^ CD3^+^ and CD8^+^ expression. Live/Dead fixable aqua dead cell stain kit (Molecular Probes) was used to discriminate live from dead cells.

### Cells and transfection

HEK293T and MDCK cells were grown in DMEM (Gibco) supplemented with 10% FCS, 2 mM L-glutamine, 0.4 mM sodium pyruvate and antibiotics. HEK293T cells were transfected using the calcium phosphate precipitate transfection method with specific expression vectors (pCAGGS-E-hA20 (LMBP 3778), pCAGGS-E-RIG-I-CARD (LMBP 6517), pEF-HA-IRF-7 (kindly provided by T. Taniguchi, Graduate School of Medicine and Faculty of Medicine, University of Tokyo)), NF-κB, IRF3, IRF7 reporter plasmids (respectively pConLuc (LMBP3248), pISRE-luc (LMBP4011), pGL3-IFNα4-luc (kindly provided by J. Hiscott, McGill University, Montreal, Quebec, Canada), and pACTbetagal (LMBP4341) for transfection efficiency normalization. Details of plasmids are presented along with detailed sequence maps at the BCCM-LMBP plasmid databank http://bccm.belspo.be/index.php.

For the generation of BMDM, bone marrow cells were cultured 7 days in RPMI 1640 (Gibco) supplemented with 10% FCS, 2 mM L-glutamine, 0.4 mM sodium pyruvate, antibiotics and 40 ng/ml recombinant M-CSF. BMDM were of ≥95% purity as measured by flow cytometry using F4/80 and CD11b specific antibodies. For the isolation of alveolar macrophages, the trachea was canulated and the lung was flushed 4 times with HBSS containing 1 mM EDTA. Alveolar macrophages were cultured in RPMI 1640 (Gibco) supplemented with 1% FCS, 2 mM L-glutamine, 0.4 mM sodium pyruvate and antibiotics.

### Western blotting

For total lysates, cells were lysed at 4°C for 15 min in lysis buffer (200 mM NaCl, 1% NP-40, 10 mM Tris-HCl pH 7.5, 5 mM EDTA, 2 mM DTT) supplemented with protease and phosphatase inhibitors. Nuclear and cytoplasmic lysates were prepared by resuspending cells in B1 (10 mM Hepes pH 7.5, 10 mM KCl, 1 mM MgCl_2_, 5% glycerol, 0.5 mM EDTA and 0.1 mM EGTA supplemented with protease and phosphatase inhibitors) for 15 min at 4°C. Next, NP-40 detergent was added to a final concentration of 0.65% and cells were centrifuged at 500 g for 5 min. The nuclear fraction containing pellet was lyzed in B2 (20 mM Hepes pH 7.5, 1% NP-40, 400 mM NaCl, 10 mM KCl, 1 mM MgCl2, 20% glycerol, 0.5 mM EDTA and 0.1 mM EGTA supplemented with protease and phosphatase inhibitors) for 15 min at 4°C. The lysates were subsequently separated by SDS-PAGE and analyzed by western blotting and ECL detection (Perkin Elmer Life Sciences). Immunoblots were revealed with anti-A20, anti-IκBα, anti-p65, and anti-histon H1 (Santa Cruz), anti-IRF3 (Invitrogen), anti-phospho-IRF3 and anti-phospho-IκBα (Cell Signaling) and anti-actin (MP Biomedicals). The density of the bands was quantified (fold induction) with the ImageJ (http://rsbweb.nih.gov/ij) Gel analyzer tool. All intensities were calculated relative to the first lane ( = time 0).

### Flow cytometry

Lungs were dissected and incubated with collagenase type IV (1 mg/ml; Sigma) and DNAse (100 U/ml; Roche) at 37°C for 30 min. Subsequently, samples were filtered through a 70 µm and 40 µm nylon mesh. For the preparation of BAL, trachea were canulated and airway lumen was flushed 4 times with HBSS with 1 mM EDTA. Cells were stained with monoclonal antibodies directed against MHC-II (I-A/I-E) FITC (M5/114.15.2), CD11c PerCP-Cy5.5 (N418), F4/80 APC (BM8), CD62L PE (MEL-14), Granzyme B FITC (NGZB) from eBiosciences and CD3 Molecular Complex Horizon v450 (17A2), Ly6C Horizon v450 (AL-21), Ly6G PE (1A8), CD11b APC-Cy7 (M1/70), CD8α PerCP (53-6.7), IFNγ Alexa 647 (XMG1.2) and CD16/32 (2.4G2) from BD Pharmingen. Samples were acquired on a LSRII Cytometer and analyzed using FACSDiva software (BD Biosciences). Propidium iodide was used to discriminate between live and dead cells.

### Cytokine quantification

For TNF ELISA, 96-well plates were coated with TNF coating (TN3-19, eBioscience) and detection (R4-6A2, eBioscience) antibodies. IFNα and IFNβ protein levels were determined with an ELISA kit (PBL Biomedical Laboratories). For IFNγ ELISA, 96-well plates were coated with IFNγ coating (XMG1.2) and detection (R4-6A2) antibodies (eBiosciences). Detection of MCP-1, KC, TNF, IL-1β and IL-6 in BAL fluid was performed using Bioplex (BioRad) technology according to the manufacturer's instructions. Milliplex technology (Millipore) was used for the detection of MIP-2 in BAL fluid.

### RNA isolation, cDNA synthesis and qPCR

Total RNA was extracted using Aurum Total RNA mini kit (BioRad) and reverse transcribed into cDNA with iScript cDNA synthesis kit (BioRad) according to the manufacturer's instructions. qPCR was performed by using SYBR Green I master mix I (Roche) in the Lightcycler 480 detection system (Roche) with the following primers: HPRT: 5′-AGTGTTGGATACAGGCCAGAC-3′ and 5′CGTGATTCAAATCCCTGAAGT-3′; IL-6: 5′-GAGGATACCACTCCCAACAGACC-3′ and 5′-AAGTGCATCATCGTTGTTCATACA-3′; IFNβ: 5′-TCAGAATGAGTGGTGGTTGC-3′ and 5′-GACCTTTCAAATGCAGTAGATTCA-3′; A20: 5′-AAACCAATGGTGATGGAAACTG-3′ and 5′-GTTGTCCCATTCGTCATTCC-3′; CCL2: 5′-TTAAAAACCTGGATCGGAACCAA-3′ and 5′-GCATTAGCTTCAGATTTACGGGT-3′; CXCL1: 5′-GAGCCTCTAACCAGTTCCAG-3′ and 5′-TGAGTGTGGCTATGACTTCG-3′ and IFNα4: 5′-TGATGAGCTACTACTGGTCAGC-3′ and 5′-GATCTCTTAGCACAAGGATGGC-3′. Primers were designed with PerlPrimer (http://perlprimer.sourceforge.net). Quantification was performed using the comparative C_T_ method (ΔΔC_T_). Results are expressed relative to HPRT values.

### Statistics

Results are expressed as the mean ± SEM. Statistical significance between groups was assessed using two-way ANOVA. The differences for *in vivo* experiments (at least 5 mice per group) were calculated using the Mann-Whitney U-test for unpaired data. Statistical significance of differences between survival rates was analyzed by comparing Kaplan-Meier curves using the log-rank test (GraphPad Prism version 5, GraphPad, San Diego, CA).

## Supporting Information

Figure S1 **A20 deficient alveolar macrophages are hyperresponsive to IAV infection.** Alveolar macrophages isolated from A20^myel-KO^ and wild type (A20^myel-WT^) mice were mock treated or infected with IAV (moi 1) for 18 hours. TNF, MCP-1 (ccl2), KC (cxcl1) and IFNα4 mRNA expression was determined by qPCR (A). Cell culture supernatant was analyzed for TNF, MCP-1 and KC protein levels by ELISA and multiplexing technologies (B). Error bars represent mean values (+/− SEM) of 2–3 samples. Results are representative for 2 independent experiments. *p<0.05; **p<0.01; ***p<0.001.
(TIF)

Figure S2 **Adaptive immunity is not altered by the absence of A20 in myeloid cells following sublethal IAV infection.** Wild type (A20^myel-WT^) and A20^myel-KO^ mice were infected intranasally with a sublethal dose of IAV. BAL was isolated from IAV infected mice at 4, 7 and 10 days p.i. and analyzed for total CD8^+^ T cells, Granzyme B (GrB) and IFNγ expressing CD8^+^ T cells (A) and IFNγ protein levels (B). Total CD8^+^ T cells, GrB and IFNγ expressing CD8^+^ T cells in the lung parenchyma were also determined by flow cytometry (C). Virus specific antibody titers in serum at 4, 7 and 10 days p.i. were determined via a hemagglutination inhibition (HAI) assay. The dashed line depicts the detection limit of the assay (D). Numbers are averages +/− SEM of at least 5 mice per group and are representative of 2 independent experiments.
(TIF)

Figure S3 **Adaptive immunity is not altered by the absence of A20 in myeloid cells following lethal IAV infection.** Wild type (A20^myel-WT^) and A20^myel-KO^ mice were infected intranasally with a lethal dose of IAV. 6 days p.i. mice were sacrificed and analyzed. (A) TNF and IL-1β protein levels in BAL fluid were measured by ELISA and multiplexing technologies. (B) Viral titers in the lung were measured and expressed as mean TCID_50_. The dashed line represents the detection limit. (C) Total CD8^+^, GrB and IFNγ expressing T cells in BAL fluid were measured using flow cytometry. (D) IFNγ protein levels in BAL fluid were measured by ELISA. (E) Total CD8^+^, GrB and IFNγ expressing T cells in lung parenchyma were measured using flow cytometry. (F) Virus specific antibody titers in serum were determined via a hemagglutination inhibition (HAI) assay. The dashed line depicts the detection limit. Numbers are averages +/− SEM of at least 5 mice per group. *p<0.05; **p<0.01.
(TIF)
