# Correction: A20 (*Tnfaip3*) Deficiency in Myeloid Cells Protects against Influenza A Virus Infection

**DOI:** 10.1371/annotation/a2136b9a-3cbf-426f-9cfb-73e3c9c6396d

**Published:** 2012-04-03

**Authors:** Jonathan Maelfait, Kenny Roose, Pieter Bogaert, Mozes Sze, Xavier Saelens, Manolis Pasparakis, Isabelle Carpentier, Geert van Loo, Rudi Beyaert

There was an error in affiliations 1, 3, and 4 for authors Jonathan Maelfait, Kenny Roose, Pieter Bogaert, Mozes Sze, Xavier Saelens, Isabelle Carpentier, Geert van Loo, and Rudi Beyaert. Affiliation 1 should be: Unit of Molecular Signal Transduction in Inflammation, Department for Molecular Biomedical Research, VIB, Ghent, Belgium. Affiliation 3 should be: Unit of Molecular Virology, Department for Molecular Biomedical Research, VIB, Ghent, Belgium. Affiliation 4 should be: Cell Culture and Sorting Core Facility, Department for Molecular Biomedical Research, VIB, Ghent, Belgium.

