# Supplementary figures and images for: A20 (Tnfaip3) Deficiency in Myeloid Cells Protects against Influenza A Virus Infection
Source: PLoS Pathog. 2012 Mar 1;8(3):e1002570. doi: 10.1371/journal.ppat.1002570 (PMC3291650; doi:10.1371/journal.ppat.1002570)

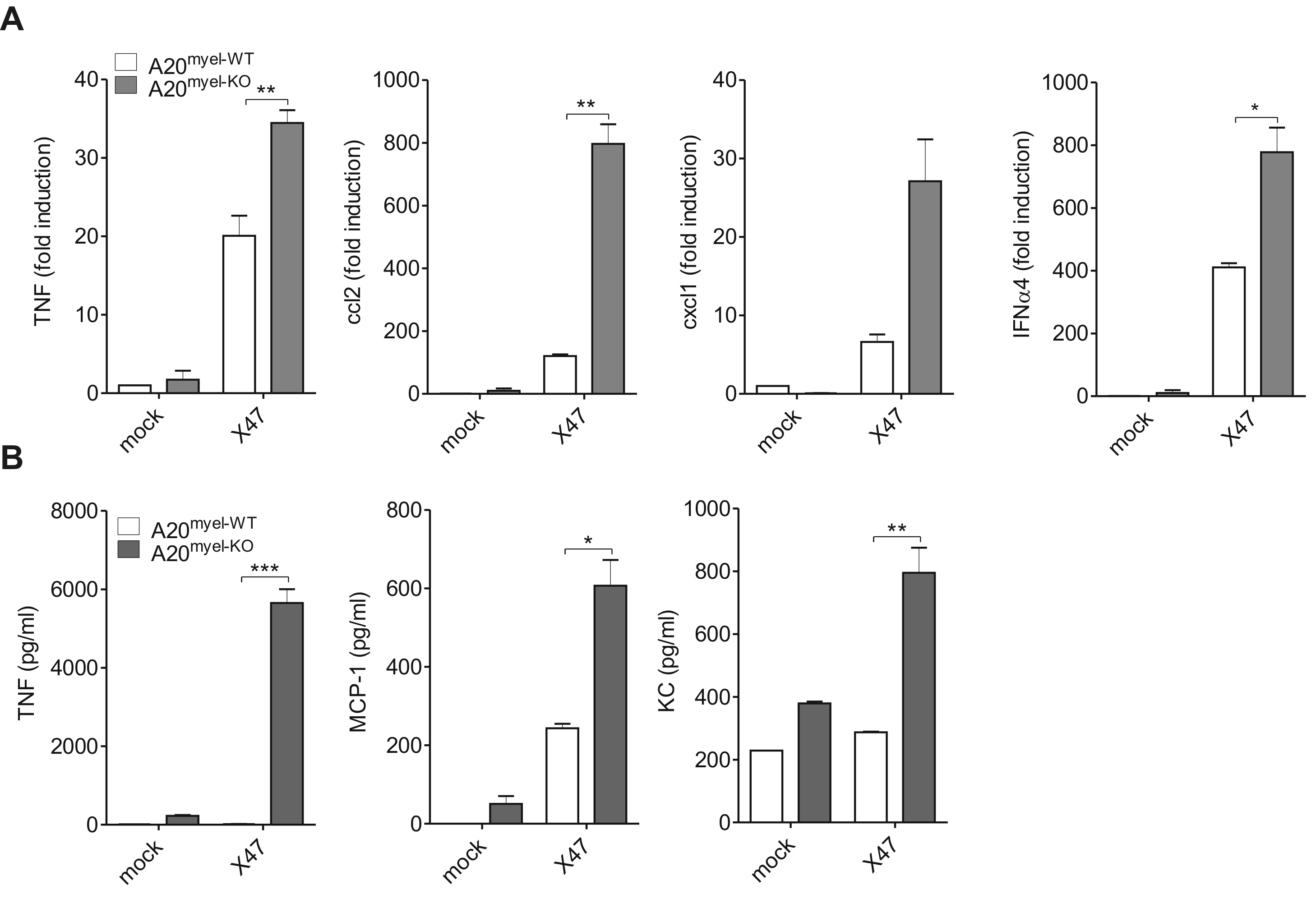

Supplement: Figure S1 — A20 deficient alveolar macrophages are hyperresponsive to IAV infection. Alveolar macrophages isolated from A20myel-KO and wild type (A20myel-WT) mice were mock treated or infected with IAV (moi 1) for 18 hours. TNF, MCP-1 (ccl2), KC (cxcl1) and IFNα4 mRNA expression was determined by qPCR (A). Cell culture supernatant was analyzed for TNF, MCP-1 and KC protein levels by ELISA and multiplexing technologies (B). Error bars represent mean values (+/− SEM) of 2–3 samples. Results are representative for 2 independent experiments. *p<0.05; **p<0.01; ***p<0.001. (TIF) [file ppat.1002570.s001.tif]

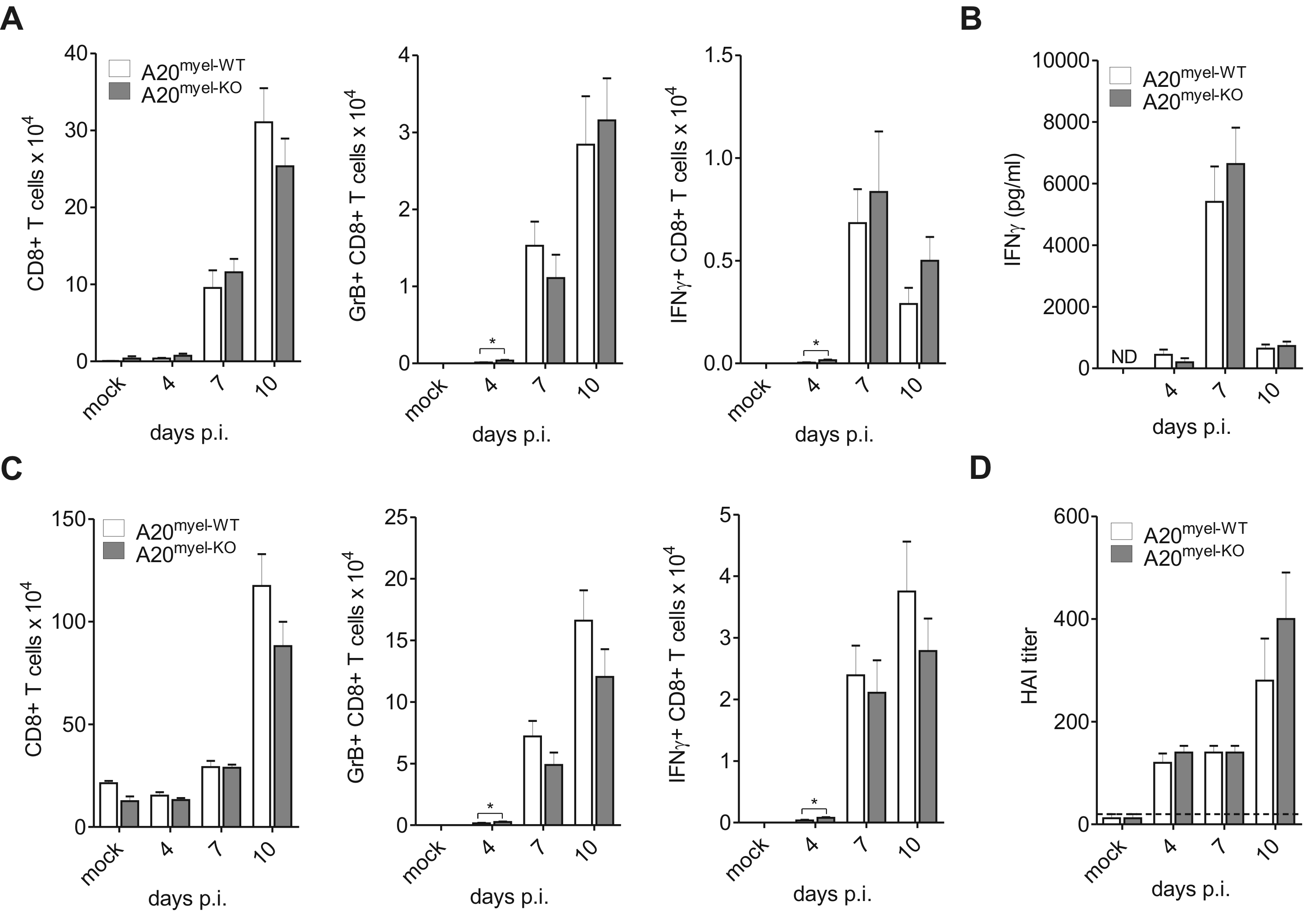

Supplement: Figure S2 — Adaptive immunity is not altered by the absence of A20 in myeloid cells following sublethal IAV infection. Wild type (A20myel-WT) and A20myel-KO mice were infected intranasally with a sublethal dose of IAV. BAL was isolated from IAV infected mice at 4, 7 and 10 days p.i. and analyzed for total CD8+ T cells, Granzyme B (GrB) and IFNγ expressing CD8+ T cells (A) and IFNγ protein levels (B). Total CD8+ T cells, GrB and IFNγ expressing CD8+ T cells in the lung parenchyma were also determined by flow cytometry (C). Virus specific antibody titers in serum at 4, 7 and 10 days p.i. were determined via a hemagglutination inhibition (HAI) assay. The dashed line depicts the detection limit of the assay (D). Numbers are averages +/− SEM of at least 5 mice per group and are representative of 2 independent experiments. (TIF) [file ppat.1002570.s002.tif]

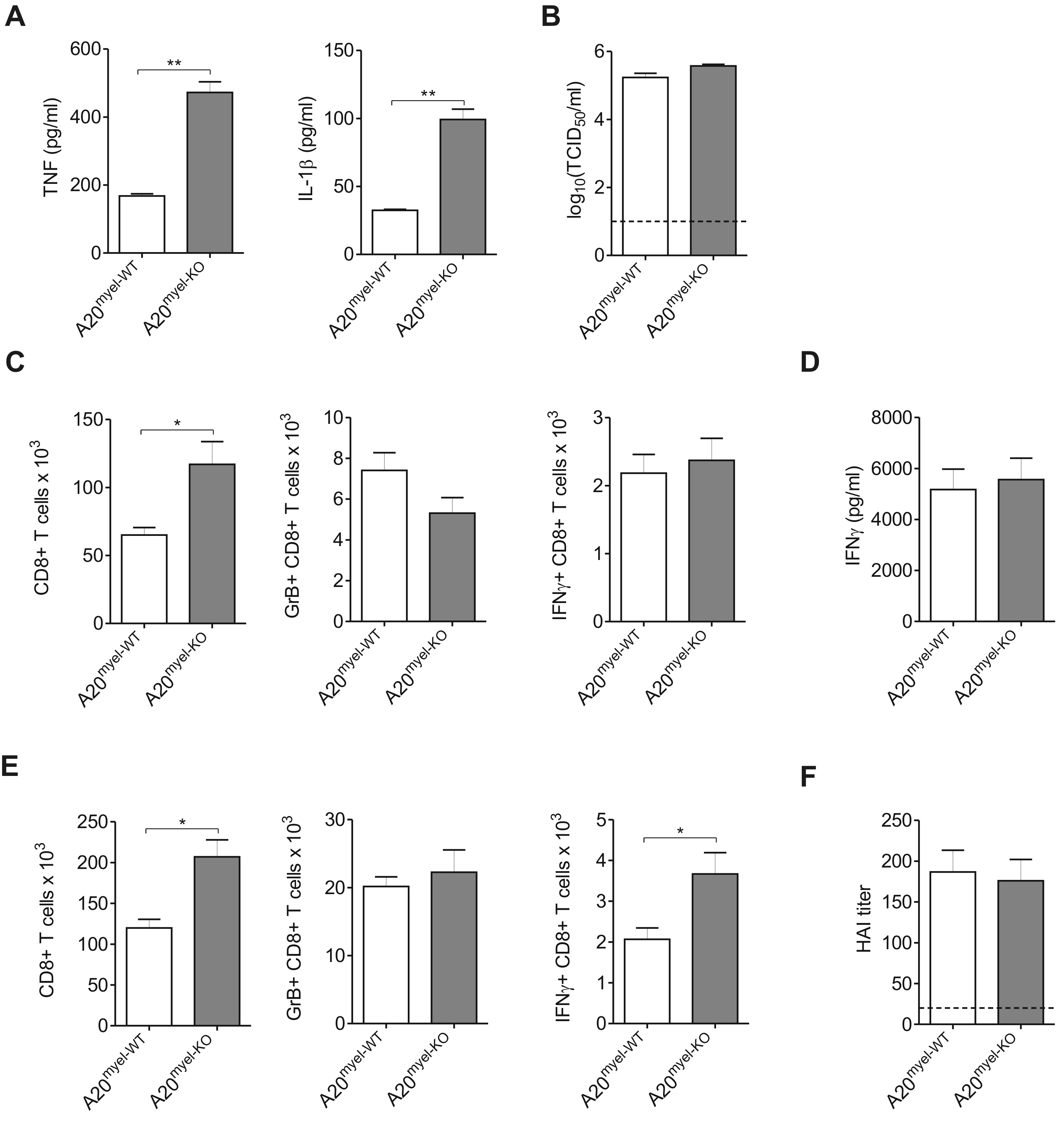

Supplement: Figure S3 — Adaptive immunity is not altered by the absence of A20 in myeloid cells following lethal IAV infection. Wild type (A20myel-WT) and A20myel-KO mice were infected intranasally with a lethal dose of IAV. 6 days p.i. mice were sacrificed and analyzed. (A) TNF and IL-1β protein levels in BAL fluid were measured by ELISA and multiplexing technologies. (B) Viral titers in the lung were measured and expressed as mean TCID50. The dashed line represents the detection limit. (C) Total CD8+, GrB and IFNγ expressing T cells in BAL fluid were measured using flow cytometry. (D) IFNγ protein levels in BAL fluid were measured by ELISA. (E) Total CD8+, GrB and IFNγ expressing T cells in lung parenchyma were measured using flow cytometry. (F) Virus specific antibody titers in serum were determined via a hemagglutination inhibition (HAI) assay. The dashed line depicts the detection limit. Numbers are averages +/− SEM of at least 5 mice per group. *p<0.05; **p<0.01. (TIF) [file ppat.1002570.s003.tif]
